# Effect of Process Parameters on the Formability, Microstructure, and Mechanical Properties of Laser-Arc Hybrid Welding of Q355B Steel

**DOI:** 10.3390/ma16124253

**Published:** 2023-06-08

**Authors:** Liping Zhang, Genchen Peng, Jinze Chi, Jiang Bi, Xiaoming Yuan, Wen Li, Lijie Zhang

**Affiliations:** 1 Hebei Key Laboratory of Heavy Machinery Fluid Power Transmission and Control, Yanshan University, Qinhuangdao 066004, China; 15094347413@163.com (L.Z.); xiaomingbingbing@163.com (X.Y.); liwen9933@126.com (W.L.); 2 Jiangsu XCMG Construction Machinery Research Institution Ltd., Xuzhou 221000, China; 3 Key Laboratory of Advanced Forging & Stamping Technology and Science, Ministry of Education of China, Yanshan University, Qinhuangdao 066004, Chinabijiang198905@163.com (J.B.)

**Keywords:** Q355B steel, laser-arc hybrid welding, welding characteristic, microstructure, mechanical properties

## Abstract

Thick plate steel structure is widely used in the construction machinery, pressure vessels, ships, and other manufacturing fields. To obtain an acceptable welding quality and efficiency, thick plate steel is always joined by laser-arc hybrid welding technology. In this paper, Q355B steel with a thickness of 20 mm was taken as the research object, and the process of narrow-groove laser-arc hybrid welding was studied. The results showed that the laser-arc hybrid welding method could realize one-backing and two-filling welding with the single-groove angles of 8–12°. At different plate gaps of 0.5 mm, 1.0 mm, and 1.5 mm, the shapes of weld seams were satisfied with no undercut, blowhole, or other defects. The average tensile strength of welded joints was 486~493 MPa, and the fracture position was in the base metal area. Due to the high cooling rate, a large amount of lath martensite formed in heat-affected zone (HAZ) and this zone exhibited higher hardness values. The impact roughness of the welded joint was almost 66–74 J, with different groove angles.

## 1. Introduction

Thick steel plate (thickness > 16 mm) is widely used in engineering machinery, pressure vessels, construction machinery, and other fields [1,2,3]. The welding quality and efficiency determine the core competitiveness of the product [4]. The traditional welding methods of thick plate steel are mainly gas-shielded welding (GSW) or submerged-arc welding (SAW), which have reduced the welding automation degree and welding efficiency and affected performance [5,6,7,8,9]. Ning et al. [7] proposed that composite welding can obviously improve the tensile strength and plasticity of high-nitrogen austenitic stainless steel joints compared with metal–inert gas (MIG) welding. Li and Zhang [8] welded high-nitrogen austenitic stainless steel by laser-arc hybrid welding technology and their results indicated there was no soft zone in the welded joint. Balakrishnan et al. [9] compared a 30 mm thick SA508 steel plate with four welding processes and found that the width of the peak longitudinal tensile residual stress area of the laser-arc hybrid weld was the largest. However, with an increase in plate thickness, the number of filling layers increases accordingly, which further leads to increases in the welding costs and time [10]. Therefore, the development of a high-efficiency and high-quality welding technology for thick steel plate is becoming a research focus at home and abroad.

In recent years, as a high-quality and efficient welding method, laser-arc hybrid welding has gradually become the focus of the international welding industry due to the broad application prospects and market competitiveness [11]. This method combines the advantages of the large penetration capacity of laser welding and the strong gap adaptability of arc welding [12]. At the same time, the filler metal of fusion electrode wire can promote the smooth transition of the welding seam, improve the gap lap ability, reduce the difficulty of workpiece assembly, and effectively improve the welding speed [13]. Zhu et al. [14] studied the double-sided laser-MIG (metal-inert gas) welding of SA553 cryogenic steel and investigated the micro-hardness and impact roughness of the welded joint. The results showed that the hardness of HAZ had the highest value of 350 HV and the impact absorbed energy of the fusion zone was 172 J. Üstündağ et al. [15] optimized the laser-arc welding parameters of X120 grade HSLA steel and found that the tensile strength of the welded joint decreased when the heat input exceed 1.6 kJ/mm, and the lowest tensile strength was 915 MPa. Lei et al. [16] joined the high-nitrogen steel by the laser-MIG method and investigated the effect of MnN power addition on the mechanical properties of the welded joint. After being modified by the MnN powder, the tensile strength and impact absorbed energy of the welded joint increased significantly to 1072 MPa and 28.5 Ak/J, respectively. Bao et al. [17] analyzed the influence of process parameters on the relationship of the microstructure and the toughness of laser-arc welded EQ70 steel. 

The coarse grain located near the HAZ consisted of lath martensite and polygonal ferrite, and the impact absorb energy of the HAZ obviously decreased. During the laser-arc welding, some other process optimizations can effectively improve the welding quality, such as laser beam oscillation, the pulse laser, and an additional energy field. The improvement in melt flow can reduce the porosity defect and refine the microstructure. Meng et al. [18] used the pulse laser to join the stainless steel and found that the porosity ratio decreased significantly. Hao et al. [19] achieved the plasticity improvement of an AZ31 Mg alloy welded joint by laser beam oscillation. In short, with a laser beam oscillation in the laser–MIG welding process, the mechanical properties of welded joints can be improved due to the decreased welding defects and microstructure optimization.

In this paper, Q355B steel with a thickness of 20 mm was joined by the laser–MIG welding method. The weld seam formation, microstructure, and mechanical properties of welded joints at different groove angles and plate gaps were investigated in detail. The research result provides a basis for the high-efficiency and high-quality welding process of thick steel plate construction.

## 2. Experimental Material and Methods

### 2.1. Materials

The base metal used for the laser–MIG hybrid welding test was Q355B steel and the size of welding test plate was 300 × 150 × 20 mm^3^. The filling wire was ER50-6 with a fixed diameter of 1.2 mm. The chemical compositions of the base metal and the filling wire are listed in Table 1. Before the laser–MIG welding process, the Q355B sheet was manufactured with a welding groove that could be joined by Y-type butt welding. The angles of the welding groove were 8° and 12° and the height of root face was 8 mm. The surface of the welding zone was cleaned by acetone to remove oil contamination.

### 2.2. Welding Process

**Figure 1:**
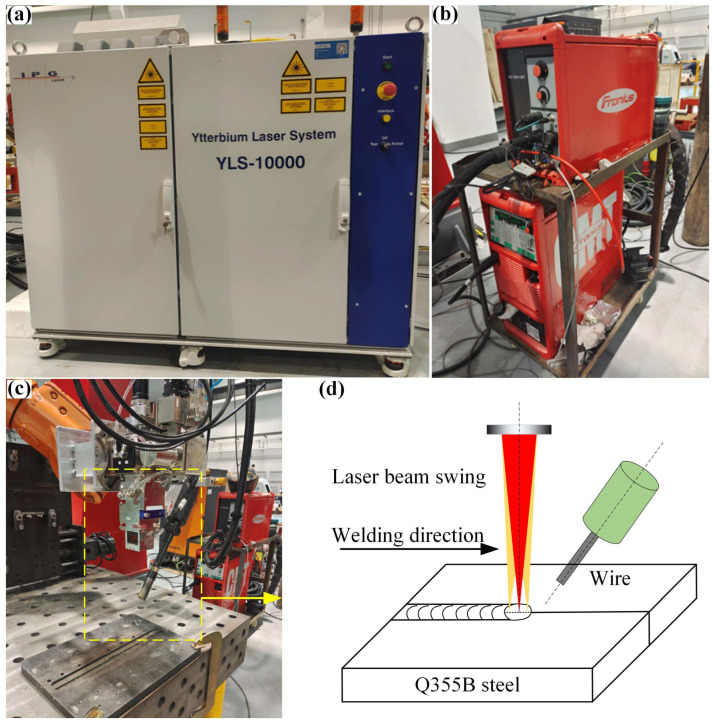
Schematic diagram of laser-arc hybrid welding of Q355B steel: (**a**) fiber laser; (**b**) CMT welding machine; (**c**) laser head and welding gun; (**d**) laser–MIG hybrid welding schematic diagram.

The laser–MIG hybrid welding system is shown in Figure 1. An IPG YSL-10000 fiber laser with a maximum output power of 10 kW was used as the power source. The laser wavelength and focal length were 1064 nm and 310 μm, respectively. The laser beam formed a minimum spot diameter of 0.15 mm after focusing. The MIG welding machine was a synergic 5000 Phoenix CMT welding machine (Fronius International GmbH, Wels, Austria) with a maximum output current of 400 A [20]. The laser head and the welding gun were fixed to a six-axis KUKA robot by professionally designed tooling, with a fixed filament spacing of 1 mm and a laser defocus amount of −1 mm. The Q355B sheets were fixed to the welding platform by the tooling fixture, and the control welding gaps were set between 0.5 mm to 1.5 mm. Three layers were deposited along the thickness direction in total. The corresponding welding parameters are listed in Table 2.

### 2.3. Microstructure and Mechanical Properties

**Figure 2:**
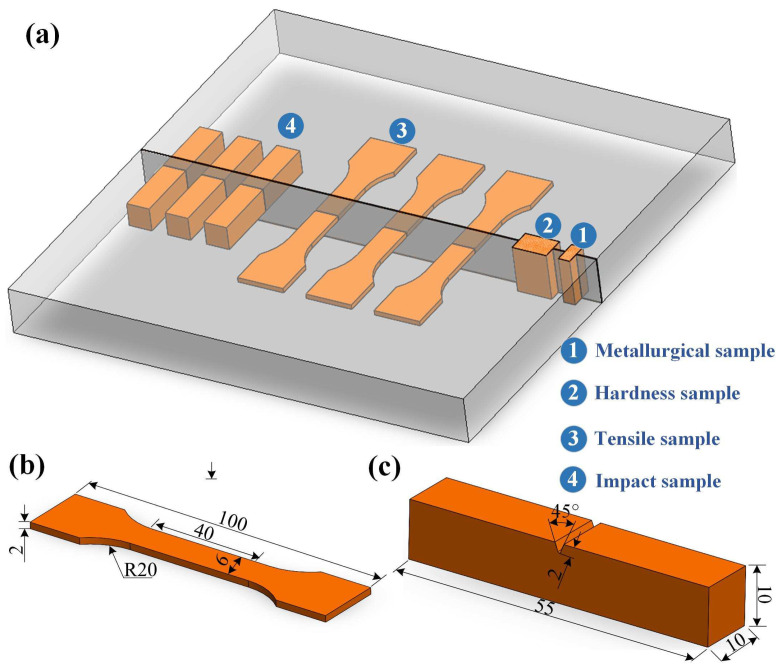
Schematic diagram of laser-arc hybrid welding of Q355B steel: (**a**) specimen extraction plan of Q355B butt-welded joint; (**b**) tensile specimen; (**c**) impact specimen.

The welded specimens were cut by a line-cutting machine, then cleaned by ultrasonic cleaning for 5 min to remove the oil stain. Four types of specimens—a metallurgical sample, a hardness sample, a tensile sample, and an impact sample—were manufactured, and the specimen sizes were determined by the standard of ISO 6892:1998 [21], as shown in Figure 2. The microstructure of the welded joint was analyzed by an optical microscope (OM). The tensile properties of the welded specimens at different welding parameters were tested on a tensile testing machine with a constant tensile speed of 2 mm/min at room temperature. The impact test was conducted by a testing machine, and the micro-hardness was measured by a Vickers hardness tester. The load and the holding time in the micro-hardness test were 500 g and 15 s, respectively.

## 3. Results and Discussion

### 3.1. Weld Formability

**Figure 3:**
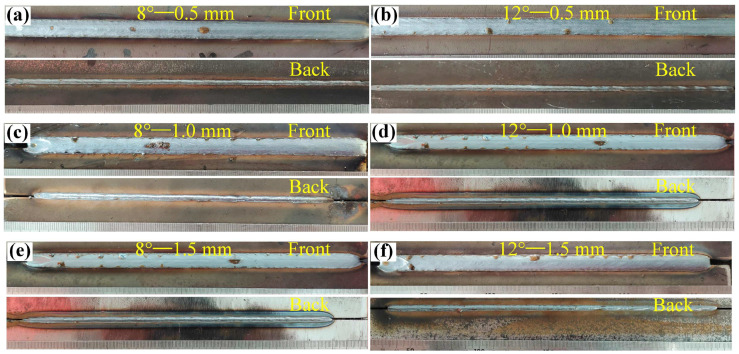
Q355B steel weld forming at different groove angles: (**a**) 8°—0.5 mm; (**b**) 12°—0.5 mm; (**c**) 8°—1.0 mm; (**d**) 12°—1.0 mm; (**e**) 8°—1.5 mm; (**f**) 12°—1.5 mm.

Figure 3 shows the weld seam formation of Q355B steel joined by the laser-arc method. Figure 3a,c,e shows the weld seam formation with a groove angle of 8°. Figure 3b,d,f shows the weld seam formation with a groove angle of 12°. The gaps of the test plates were 0.5 mm, 1.0 mm, and 1.5 mm, respectively. Some spherical splash can be observed on the top surface of the welded joint, and the undercut defects were mitigated with he increases in the plate gap. With groove angles of 8~12° and plate gaps of 0.5~1.5 mm, the laser–MIG welded joints of Q355B steel had an acceptable weld-forming quality. For the selected process parameters, no discontinuous weld seam forming occurred, and the front and back surfaces of the welded joint had good forming quality. During the laser–MIG welding, the energy of the laser beam and the electric arc were coupled together to obtain a better welding result of 1 + 1 > 2 [22,23,24]. In the welding process of thick steel plate, the assembly gap tolerance of laser-arc welding is not as strict as that of single-laser welding. Therefore, the laser-arc welding method exhibited high adaptability for the joining of Q355B steel.

**Figure 4:**
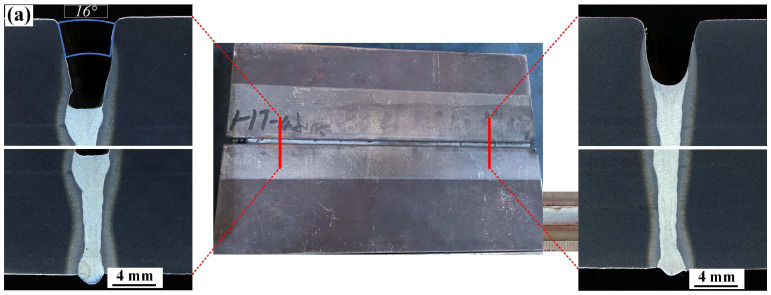

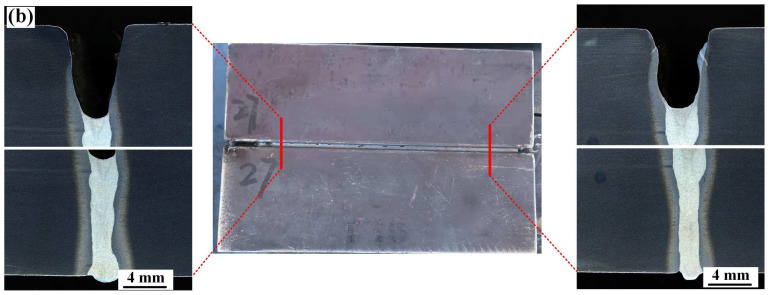
The first filling layer of Q355B steel at different gaps: (**a**) 0.5 mm and (**b**) 1.0 mm.

A cross-section of the first laser–MIG weld filling layer is shown in Figure 4. With the increase in the plate gap, the weld width increased gradually. When the plate gap was 1.0 mm, the weld width from top to bottom was more uniform. For the 0.5 mm plate gap, the spreadability of the melt pool decreased due to the unsuitable gap value. In Figure 4a, several gas pore defects with small sizes are shown at the bottom of the filling layer, indicating that the molten pool flow behavior was uneven. Moreover, the fusion line was slightly changed with the increase in the plate gap value. For multi-layer welding, the welding quality of the previous layer directly affects the metal spreading behavior of the following layer, which further affects the welding quality [25]. Therefore, to obtain a better welded joint, the first layer must have uniform fusion behavior due to the suitable process parameter. With a plate gap of 1.0 mm, the first layer had a better welding quality than it did with a plate gap of 0.5 mm.

### 3.2. Microstructure Analysis

**Figure 5:**
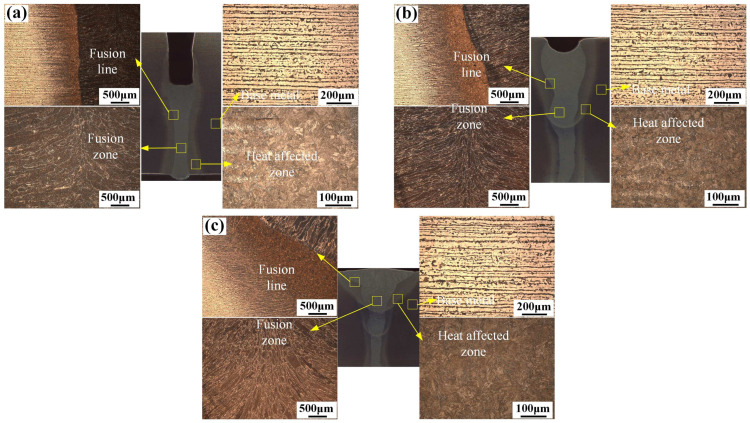
The microstructure of Q355B welded joint at different layers: (**a**) first layer; (**b**) second layer; (**c**) third layer.

At a constant plate gap of 1.0 mm, the microstructures of the fusion line, the base metal (BM), the fusion zone, and the HAZ are shown in Figure 5. Figure 5a–c shows the microstructures of the first layer, the second layer, and the third layer, respectively. For different layers, the microstructure of corresponding zone was almost the same. The BM exhibited a common microstructure morphology of a rolling state. The fusion zone had a mixed structure of columnar and equiaxed grains, where the columnar grains grew from the fusion line to the center area and the equiaxed grains formed at the middle of the fusion zone. Compared with the fusion zone of the first layer, the second layer, and the third layer, the columnar crystals grew more and more densely along the fusion line with the increase in the number of layers. The HAZ showed different morphologies compared with the fusion zone and the base metal. Due to the heat input of the laser and the arc hybrid source, the grain structure at the HAZ was a bit more than that of the BM [26,27,28].

**Figure 6:**
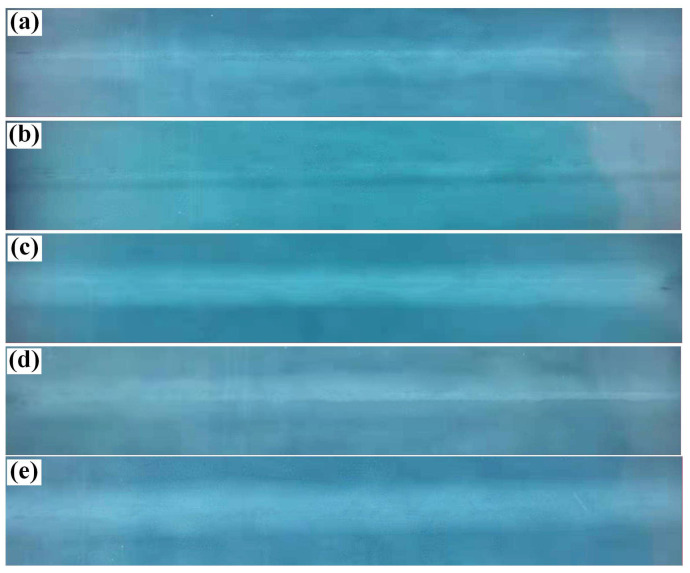
The X-ray detection of Q355B steel laser-arc welded joint: (**a**) 8°—0.5 mm; (**b**) 8°—1.0 mm; (**c**) 8°—1.5 mm; (**d**) 12°—1.0 mm; (**e**) 12°—1.5 mm.

The X-ray detection results of Q355B steel joints welded at different process parameters are shown in Figure 6. Almost no welding defects, such as non-fusion and inclusion, were observed in the whole weld seam, except that a few welding defects formed at the beginning and end of the weld seam. Due to the laser beam oscillation effect, the flow of the melt pool can be accelerated by the Marangoni convection. In addition, the pores escaped rapidly from the molten pool. Based on a statistical calculation of the pores, the porosity ratio of the welded joint was less than 2%. On one hand, the fluidity of the molten pool was enhanced by laser beam oscillation. On the one hand, the fusion quality of the weld was improved and it was conducive to the elimination of porosity. With a greater decrease in porosity defects than could be achieved by the other welding method, the mechanical properties of the welded joint increased as well.

**Figure 7:**
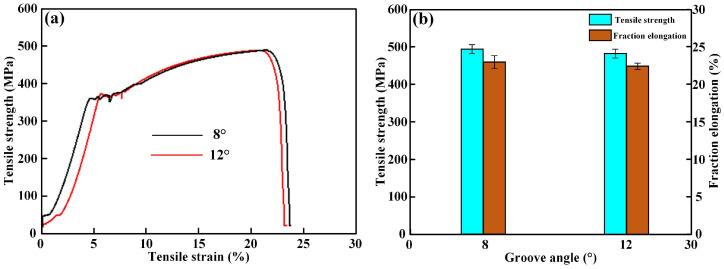
The tensile properties of Q355B steel laser-arc hybrid welding joint: (**a**) tensile stress–strain curves; (**b**) strength and elongation.

The tensile properties of Q355B steel welded by laser–MIG technology are shown in Figure 7. With a constant plate gap of 1.0 mm, the tensile stress–strain curves at the groove angles of 8° and 12° are exhibited in Figure 7a. A comparison of tensile strength and fraction elongation is shown in Figure 7b. With a small groove angle of 8°, the tensile strength and the fraction elongation of the Q355B steel welded joint were 493 MPa and 23.8%, respectively. However, as the groove angle increased to 12°, the tensile properties of the welded joint dropped slightly. When the groove angle was 12°, the tensile strength and the fraction elongation of the welded joint were 486 MPa and 22.9%, respectively. Due to the hybrid heat source of laser and arc, the Q355B steel joint had a better welding quality than that of arc welding. The welding defects such as porosity and poor weld formation were the main factors that affected the mechanical properties of the joints. However, with a suitable laser-arc process parameter and the laser beam oscillation effect, the weld seams had a good formation and the melt pool had a better spread-out characteristic, which is beneficial in eliminating welding defects.

**Figure 8:**
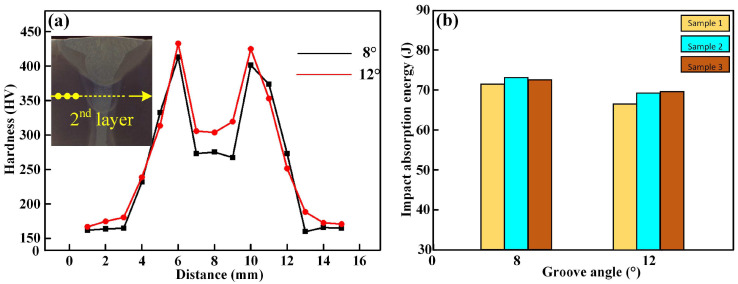
The micro-hardness distribution of the second filling layer and the impact toughness of the Q355B steel laser-arc welded joint: (**a**) micro-hardness hardness curves; (**b**) impact toughness.

The micro-hardness distribution of the second filling layer and the impact toughness of the Q355B steel laser-arc welded joint are shown in Figure 8. In Figure 8a, the micro-hardness distribution of the cross-section of the third filling layer was different with different groove angles of 8° and 12°. The micro-hardness distribution of the fusion zone exhibited as an “M” shape, and the base metal had the lowest hardness of 160~165 HV. The welded joint zone had higher hardness (270~440 HV) than the base metal. However, a higher hardness was found in the HAZ near the weld fusion line, as a large amount of lath martensite formed due to the high cooling rate. The 12° groove angle specimen at the same position ha a higher hardness due to the larger amount of filling metal. The impact absorption energy of the Q355B welded joint at different groove angles of 8° and 12° is shown in Figure 8b. For the welded joint with a groove angle of 8°, the impact absorption energies were 71~74 J. With a groove angle of 12°, the impact absorption energies of the welded joint decreased slightly, to 66~69 J. Based on the comprehensive analysis of hardness and impact toughness, it can be concluded that the higher the micro-hardness distribution of the welded joint, the lower the impact toughness.

## 4. Conclusions

(1) Twenty mm thick Q355B steel plates were successfully joined by the laser–MIG hybrid welding method, and the laser-arc welded joints of Q355B steel had an acceptable nugget form with groove angles of 8~12° and plate gaps of 0.5~1.5 mm. For the selected process parameters, the front and back surfaces of the welded joint had good forming quality.
(2) Based on the microstructure analysis of the welded joint, the fusion zone had a mixed structure of columnar and equiaxed grains, where the columnar grains grew from the fusion line to the center area and the equiaxed grains formed in the middle of the fusion zone. The HAZ showed a different morphology compared with the fusion zone and the base metal—namely the typical coarse grain morphology.
(3) The tensile strength and the elongation to fracture of the Q355B steel welded joint were 493 MPa and 23.8%, respectively, with a small groove angle of 8°. As the groove angle increased to 12°, the tensile properties of the welded joint dropped slightly. The tensile strength and the fraction elongation of the welded joint with the groove angle of 12° were 486 MPa and 22.9%, respectively.
(4) The micro-hardness distribution of the fusion zone exhibited as an “M” shape, as the base metal had the lowest hardness of 160~165 HV. The fusion zone had a higher hardness, of 270~440 HV, than that of the base metal. The impact absorption energies at the center of the fusion zone were 66~74 J.

## Figures and Tables

**Table 1:** The chemical composition of Q355B steel and filling wire used for the laser–MIG welding (wt.%).

Materials	C	Si	Mn	P	S	Cu	Cr	Ni	Fe
Q355B	0.18	0.50	1.55	0.025	0.025	0.30	0.30	0.30	Bal.
ER50-6	0.08	0.87	1.45	0.012	0.013	0.125	0.031	0.017	Bal.

**Table 2:** The welding process parameters of laser-arc welding of Q355B steel.

Groove Angle	Layer	Parameters		
		Laser Power (kW)	Current (A)	Welding Speed (m/min)
8°	Root	6.5	200	1.0
	Filling	1.5	240	0.6
	Filling	1.5	260	0.4
12°	Root	7.0	190	1.0
	Filling	1.5	240	0.6
	Filling	1.5	280	0.4

## Data Availability

The experimental data that support the findings of this study are available from the corresponding authors upon request.
